# Effects of Resistance Training on Pain, Muscle Strength, and Function in Patients Undergoing Total Knee Arthroplasty: A Systematic Review and Meta-Analysis

**DOI:** 10.3390/jcm14144979

**Published:** 2025-07-14

**Authors:** Jaehyun Lim, Byeonggeun Kim

**Affiliations:** 1 Department of Physical Therapy, Graduate School, Nambu University, Gwangju 62271, Republic of Korea; 2 Regional Health & Medical Center for Persons with Disabilities, Chonnam National University Hospital, Gwangju 61469, Republic of Korea

**Keywords:** total knee arthroplasty, resistance training, pain, muscle strength, function

## Abstract

**Background/Objectives**: The importance of resistance training for functional recovery in Total Knee Arthroplasty (TKA) patients has been emphasized. Therefore, this systematic review and meta-analysis was conducted to analyze its effects on pain, muscle strength, and function in patients with TKA. **Methods**: The following databases were used: PubMed, Web of Science, the Cochrane Library, and Embase. Randomized controlled trials that administered resistance training to patients undergoing TKA and measured pain, strength, and function were included. The risk of bias was assessed using the Cochrane Risk of Bias 2.0 tool. Effect sizes were calculated using Hedges’ g and are presented as Standardized Mean Differences (SMDs) with 95% Confidence Intervals (CIs). Subgroup analyses were conducted to determine the effect size based on the type and duration of the intervention. **Results**: The study selection process resulted in the inclusion of seven studies comprising a total of 439 participants. The bias assessment found that three studies had a low risk of bias and four had some concerns. Resistance training was effective in improving pain (SMD: 0.84, 95% CI: 0.11; 1.57, I^2^: 89.6%), muscle strength (SMD: 1.03, 95% CI: 0.29; 1.77, I^2^: 83.1%), self-reported function (SMD: 1.58, 95% CI: 0.15; 3.01, I^2^: 93.1%), and performance-based function (SMD: 0.74, 95% CI: 0.38; 1.11, I^2^: 68.9%). Subgroup analysis revealed significant differences in pain, strength, and performance-based function by comparison group, performance-based function by intervention duration, and self-reported function by intervention type. **Conclusions**: Resistance training improves pain, muscle strength, and function in TKA patients. Additionally, resistance training appears particularly effective when implemented as a standalone intervention or for durations under 12 weeks. These findings suggest that the design of resistance training protocols should be considered in clinical practice.

## 1. Introduction

Osteoarthritis (OA) is one of the most common musculoskeletal diseases. It causes severe disability, particularly in weight-bearing knee joints; surgical intervention is often required due to pain and stiffness [1]. Total Knee Arthroplasty (TKA) is a common surgery for patients with end-stage OA. Over 100,000 cases are performed each year in the UK and 700,000 cases are performed each year in the US. This number is expected to increase in the future [2]. TKA is the most clinically and cost-effective method for maintaining patients’ long-term health and functional status [3]. However, complications such as instability, stiffness, and disruption of the extensor mechanism may occur after surgery, so postoperative management is important [4].

In particular, the strength of the quadriceps femoris muscle decreases over the course of several months following TKA surgery [5]. Quadriceps femoris strength after TKA surgery is a key factor that positively affects patient expectations, symptoms, satisfaction, and functional activities [6]. In particular, it is closely related to functional outcomes such as functional mobility, stair climbing, quality of life, and activities of daily living [7]. Therefore, interventions such as preserving motor control, improving nutrition, and strengthening muscles are necessary after TKA surgery [5]. Among these interventions, resistance training has been reported to effectively strengthen muscles [8,9]. The American Physical Therapy Association recommends that TKA patients begin resistance exercise within seven days of surgery, and that it be performed gradually to improve strength, function, and range of motion [10].

Accordingly, the importance of resistance training has been increasingly emphasized for patients who have undergone TKA, and meta-analyses are continuously being conducted to analyze its effects [11,12,13]. However, previous meta-analyses included studies that implemented interventions or measurements prior to TKA surgery. Moreover, there remains a lack of studies that compare the effects of different intervention durations, as well as the single and multicomponent effects of resistance training. Therefore, this systematic review and meta-analysis aimed to evaluate the effects of resistance training on pain, muscle strength, and function in patients after TKA surgery, and to explore differences according to intervention duration and type through subgroup analysis.

## 2. Materials and Methods

This systematic review and meta-analysis was conducted in accordance with the PRISMA 2020 guidelines and the Cochrane Handbook for Systematic Reviews of Interventions [14,15]. Additionally, the study was prospectively registered in PROSPERO prior to its commencement (CRD42025640995).

### 2.1. Eligibility Criteria and Exclusion Criteria

The study inclusion criteria were selected using the PICOS method. Patients: Patients who underwent TKA surgery; Intervention: Inclusion of resistance or strength training in the intervention protocol; Comparison: No-intervention, usual care, or comparator without resistance or strength training in the protocol; Outcome: Pain, muscle strength, performance-based function, and patient-reported function; Study: Randomized clinical trials (RCTs). Studies were excluded from the analysis if they met any of the following criteria: Presence of other diseases after TKA, lack of information on resistance or strength training in the intervention protocol, absence of the full text, or non-English language version.

### 2.2. Search Strategy

The data search was conducted on 22 January 2025. The databases used were PubMed, Web of Science, the Cochrane Library, and Embase. The search included studies from database inception to 22 January 2025. The search terms were “Total knee arthroplasty”, “Resistance training”, and “Strength training”. The Supplementary Material includes detailed search strategies (Table S1). In addition, the reference lists of the included studies were reviewed to identify further relevant studies. Two independent reviewers (J.-H.L. and B.-G.K.) conducted the study selection. Any disagreements were resolved through discussion and consensus.

### 2.3. Data Collection

The retrieved studies were managed using EndNote 2018, and the collected data were organized using Excel 2022. The collected data items were the study title, year of publication, number of participants, sex, age, Body Mass Index (BMI), the intervention method of the experimental and comparison groups, the number of sessions, exercise intensity, outcome, and the pre- and post-intervention mean and standard deviation values.

To prevent overestimating the effect size when outcomes of the same category exist, we collected them in a prioritized manner. For pain, we prioritized structured tools such as the Western Ontario and MacMaster Universities Osteoarthritis Index (WOMAC) and the Knee Injury and Osteoarthritis Outcome Score (KOOS) over simple measures such as Visual Analogue Scale (VAS). For strength, we prioritized the strength of the quadriceps femoris muscle. Function collected self-reported and performance-based outcomes. The self-reported function included tools to assess Activities of Daily Living (ADL), such as the WOMAC function, the KOOS ADL, and the Hospital for Special Surgery (HSS) knee score. Two independent reviewers (J.-H.L. and B.-G.K) conducted the study selection and data extraction. Any discrepancies were resolved through discussion and consensus.

### 2.4. Assessment of Risk of Bias and Certainty of Evidence

The risk of bias in the included studies was assessed using the Cochrane Risk of Bias 2 (ROB 2) tool. The ROB 2 tool assesses five domains: The randomization process, deviations from intended interventions, missing outcome data, measurement of the outcome, and selection of the reported result. In addition, the certainty of evidence for the overall pooled results was evaluated using the GRADE (Grading of Recommendations, Assessment, Development and Evaluations) approach, which considers five factors: Risk of bias, inconsistency, indirectness, imprecision, and publication bias. Based on these domains, the overall certainty of evidence was rated as high, moderate, low, or very low [16]. Based on these domains, the overall certainty of evidence was rated as high, moderate, low, or very low. Two independent reviewers (J.-H.L. and B.-G.K) conducted the risk of bias and certainty of evidence assessments and compared the results. Any discrepancies were resolved through discussion and consensus.

### 2.5. Statistical Analysis

A meta-analysis was conducted using R Studio 4.3.1 software. Since all collected outcomes were continuous and the characteristics of the participants and intervention methods were diverse, a random-effects model with restricted maximum likelihood (REML) estimation was selected. The mean difference was calculated using the pre- and post-intervention means and standard deviations of the experimental and comparison groups. Since the same outcome was assessed using different instruments across studies, the effect sizes were expressed as Standardized Mean Differences (SMDs). The effect size was calculated using Hedges’ g. A small effect size is 0.3, a medium effect size is 0.5, and a large effect size is 0.8 [17]. The results are presented as SMDs and 95% Confidence Intervals (CIs) via forest plots for visual analysis. Heterogeneity between studies was assessed using both the I^2^ statistic and Cochran’s Q test. I^2^ values of 25%, 50%, and 75% were interpreted as indicating low, moderate, and high heterogeneity [18], respectively, and a *p*-value less than 0.05 was considered statistically significant.

Subgroup analyses were conducted based on comparison group, intervention duration, and type. The comparison group was categorized as either no-intervention or other intervention to examine whether the type of control condition influenced the treatment effects. The duration was categorized as less than 12 weeks or 12 weeks or more to examine whether the duration of the intervention influenced the effect size. The intervention type was classified as either resistance training alone or multicomponent training to compare the effects of resistance training alone versus in combination with other components. These subgroupings were used to further explore the differential effects of intervention characteristics. Egger’s regression analysis and funnel plots were not used to assess publication bias, as fewer than 10 studies were included. A sensitivity analysis was performed to assess whether exclusion of a study with an outlying effect size would substantially affect the overall results.

## 3. Results

### 3.1. Research Selection Process

**Figure 1:**
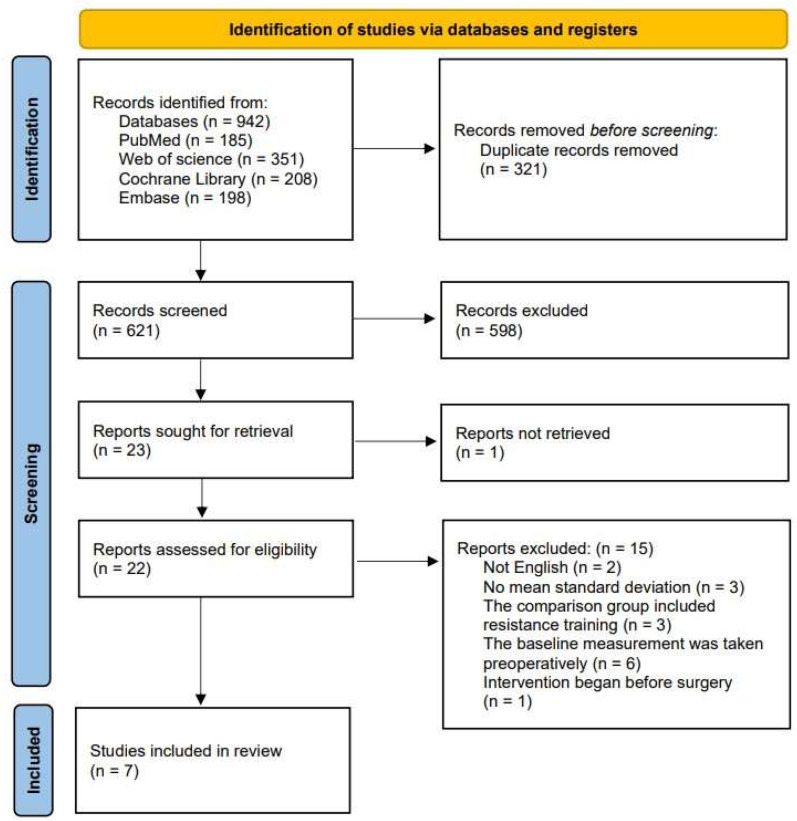
Research selection process.

A total of 942 studies were retrieved, including 165 from the PubMed database, 351 from the Web of Science database, 208 from the Cochrane Library database, and 198 from the Embase database. After removing 321 duplicates from the initial 621 records, 598 studies were excluded during title and abstract screening, and one study was excluded because the full-text report was not retrievable. As a result, 22 studies were selected for full-text review. After reading the original texts, 15 studies were excluded because they did not meet this study’s selection criteria. As a result, seven studies were included in the final analysis [19,20,21,22,23,24,25] (Figure 1).

The reasons for exclusion included not being written in English, lack of standard deviation data, inclusion of resistance training in the comparison group, baseline measurements taken preoperatively, and interventions applied before surgery [26,27,28,29,30,31,32,33,34,35,36,37,38,39,40]. The Jiao et al. study [40], which investigated high-intensity progressive training in TKA patients, was excluded because the strength training was conducted preoperatively. The reasons for excluding other papers are provided in Supplementary Table S2.

### 3.2. General Characteristics of Included Studies

A total of 439 participants were included across seven studies. The interventions applied included elastic band exercises, machine-based resistance training, aquatic resistance training, and isometric strength training. The intervention duration ranged from four weeks to one year. Various intensities were applied, including moderate intensity, One Repetition Maximum (1RM), and repetitions per set. The collected outcomes included pain, which was assessed using the WOMAC, the KOOS, VAS, and activity-specific pain. Muscle strength assessment focused on knee extensor strength. Self-reported function was assessed using the WOMAC Function, KOOS ADL, and the HSS knee score. Performance-based function was measured using the 40 m brisk walking test, the 8 ft up-and-go test, gait speed, maximal walking speed, and maximal velocity (Table S3).

### 3.3. Risk of Bias in Studies

Seven studies were assessed for bias using ROB 2. The randomization process was assessed as low risk in five studies and as having some concerns in two studies. The effect of intervention assignment was assessed as low risk in three studies and as having some concerns in four studies. Missing outcome data was assessed as low risk in four studies and as having some concerns in three studies. The measurement of the outcome was assessed as low risk in seven studies. The reported results were assessed as low risk in six studies and as having some concerns in one study. Overall, three studies were assessed as low risk, and four studies were assessed as having some concerns (Figure S1).

### 3.4. Meta-Analysis Results

#### 3.4.1. Pain

**Figure 2:**
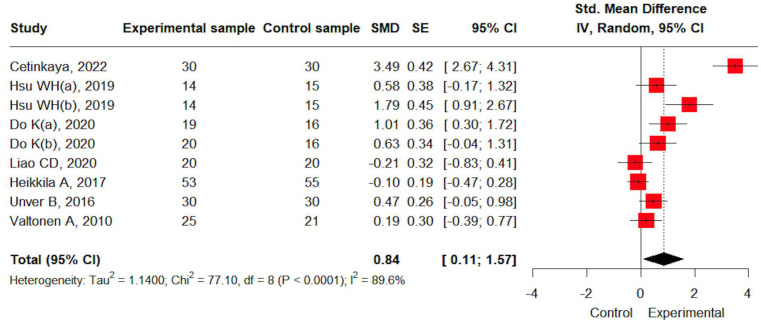
The effects of resistance training on pain [19,20,21,22,23,24,25].

Pain data were available from seven studies. A large effect size was observed for pain improvement through resistance training (SMD: 0.84, 95% CI: 0.11; 1.57). Heterogeneity was high (I^2^ = 89.6%) and statistically significant (*p* < 0.05) (Figure 2).

#### 3.4.2. Muscle Strength

**Figure 3:**
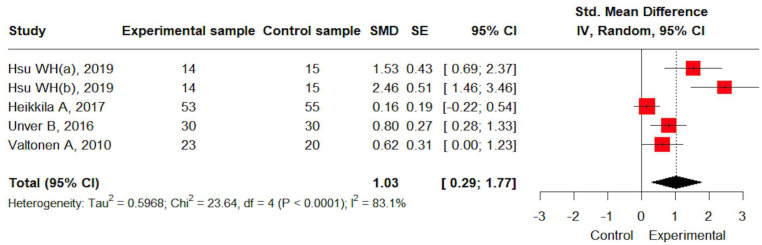
The effects of resistance training on muscle strength [20,23,24,25].

Muscle strength data were available from four studies. A large effect size was observed for strength improvement through resistance training (SMD: 1.03, 95% CI: 0.29; 1.77). Heterogeneity was high (I^2^ = 83.1%) and statistically significant (*p* < 0.05) (Figure 3).

#### 3.4.3. Self-Reported Function

**Figure 4:**
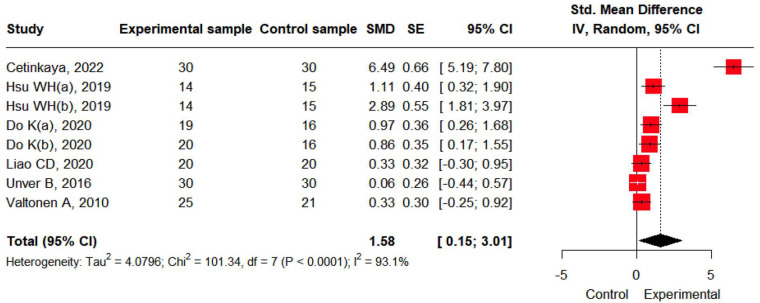
The effects of resistance training on self-reported function [19,20,21,22,24,25].

Self-reported function data were available from six studies. A large effect size was observed for self-reported functional improvement through resistance training (SMD: 1.58, 95% CI: 0.15; 3.01). Heterogeneity was high (I^2^ = 93.1%) and statistically significant (*p* < 0.05) (Figure 4).

#### 3.4.4. Performance-Based Function

**Figure 5:**
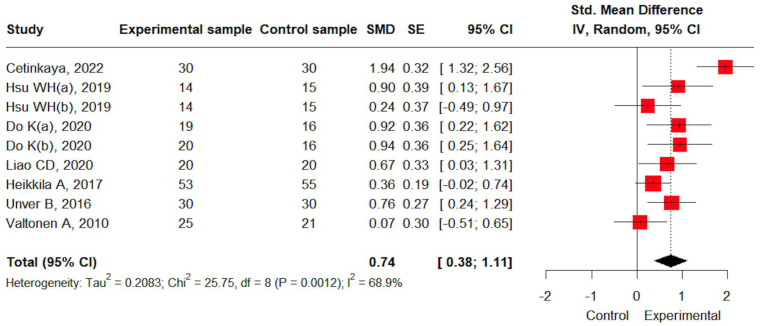
The effects of resistance training on performance-based function [19,20,21,22,23,24,25].

Performance-based function data were available from seven studies. A moderate effect size was observed for performance-based functional improvement through resistance training (SMD: 0.74, 95% CI: 0.38; 1.11). Heterogeneity was moderate (I^2^ = 68.9%) and statistically significant (*p* < 0.05) (Figure 5).

### 3.5. Subgroup Analysis

Subgroup analysis was conducted based on comparison group, intervention duration, and intervention type. Significant effects were observed in the other intervention group for all outcomes, whereas no significant effects were found in the no-intervention group. Subgroup differences were statistically significant for pain, strength, and performance-based function (*p* < 0.05), but not for self-reported function (*p* > 0.05). For intervention duration, pain showed a significant effect when the duration was less than 12 weeks, while strength and performance-based function showed significant effects regardless of duration. A significant subgroup difference was found only for performance-based function (*p* < 0.05). Subgroup differences for pain, strength, and self-reported function were not statistically significant (*p* > 0.05). Regarding intervention type, resistance training alone showed significant effects for strength and self-reported function, whereas multicomponent interventions did not. A significant subgroup difference was observed only for self-reported function (*p* < 0.05). Detailed results are shown in Table 1.

### 3.6. Sensitivity Analysis

Due to the high heterogeneity of the study results, a sensitivity analysis was conducted. Additional analyses were conducted after excluding the study by Çetinkaya [17], due to its high heterogeneity in effect sizes. As a result, significant effects were found on pain (SMD: 0.48, 95% CI: 0.07; 0.89, I^2^: 79.2%), self-reported function (SMD: 0.86, 95% CI: 0.24; 1.47, I^2^: 77.2%), and performance-based function (SMD: 0.56, 95% CI: 0.33; 0.79, I^2^: 14.5%).

### 3.7. Certainty of Evidence

The certainty of the evidence was assessed using the GRADE approach. The certainty was rated as moderate for pain, strength, and performance-based function due to serious risk of bias and inconsistency, but with no serious concerns about indirectness or imprecision. For self-reported function, the certainty was rated as low because of very serious inconsistency, serious imprecision, and serious risk of bias (Table S4).

## 4. Discussion

This systematic review and meta-analysis suggests that resistance training may lead to improvements in pain, muscle strength, and functional outcomes (WOMAC, KOOS, HSS knee score, and gait speed) among patients undergoing TKA. Additionally, subgroup analyses revealed that the effects were more pronounced when the interventions lasted fewer than 12 weeks and when resistance training was the only type of exercise performed. However, heterogeneity was high, and the risk of bias assessment showed that three studies were at low risk and four had some concerns, indicating that the overall findings were favorable but should be interpreted with caution.

A previous meta-analysis reported that active resistance exercise of the lower limbs was positively associated with pain relief, recovery of muscle strength, improved mobility, and enhanced physical function in patients who underwent TKA [11]. Another meta-analysis reported that strength training improved the distance covered in the six-minute walking test in patients with TKA [12]. In contrast, a meta-analysis with conflicting findings reported that, compared to standard rehabilitation, progressive resistance training did not significantly change functional capacity or muscle strength in patients with TKA or total hip arthroplasty [13]. The observed variations in outcomes are hypothesized to be attributable to disparities in the characteristics of the subjects included in the study and the outcomes collected. Only TKA patients were included in this meta-analysis. To ensure consistency in effect size calculation when multiple outcomes were available, subitems of the WOMAC and KOOS and walking speed were prioritized for inclusion. This may explain why the results differed from those of previous studies.

Resistance training contributes to pain relief through physiological mechanisms, including protein remodeling, reduced relative load, and decreased pain sensitivity via central inhibitory mechanisms [41,42,43]. Additionally, weakness in the quadriceps femoris negatively affects the long-term prognosis and functional recovery of TKA patients. Incorporating resistance training into the intervention program effectively improves physical function [44]. Following TKA surgery, lower extremity muscles such as the quadriceps femoris and biceps femoris lose 50–60% of their preoperative strength. This loss results in a decline in patients’ functional mobility and ability to perform activities of daily living, such as rising from a chair, stair climbing, and walking at an adequate speed [45]. Walking speed is a critical outcome for TKA patients, strongly influenced by quadriceps femoris strength [46].

Between comparison groups, statistically significant subgroup differences were observed for pain, strength, and performance-based function, with the other intervention group showing more effective outcomes compared to the no-intervention group. For self-reported function, the effect size was also somewhat larger in the other intervention group, although this difference was not statistically significant. When analyzing studies in this review that compared resistance training to no intervention, Heikkilä et al. [23] reported that it was difficult to control the exercise load in a home-based program, and participant compliance declined during the final month. Valtonen et al. [25] reported that the participants’ favorable baseline condition may have limited the potential for additional improvements, and that the training intensity was difficult to define because it was unclear whether the participants had actually reached fatigue, despite being instructed to exercise at maximum effort. These results suggest that the intensity and control of resistance training could potentially affect the effectiveness of the intervention. A study analyzing the dose–response relationship of resistance training in adults found that setting the intensity at 50–69% of 1RM produced the greatest improvement in symptoms, function, and lower extremity muscle strength [47]. In contrast, some studies found no significant difference between high- and low-intensity training. [48]. To improve strength, it is necessary to consider various training variables such as intensity, number of sets, and training frequency, as their combination can influence the effectiveness of the intervention [49]. Therefore, more high-quality studies are needed to clarify the causes of these conflicting results and understand the effects of resistance training.

A statistically significant subgroup difference by intervention duration was found for performance-based function, with greater effects seen in interventions lasting less than 12 weeks. Similar trends were noted for the other outcomes, although these differences did not reach statistical significance. These findings regarding intervention duration in the current meta-analysis differ from those of a previous meta-analysis [12], which reported that increasing the duration and frequency of resistance training improved gait and functional outcomes in patients with TKA. This discrepancy may be attributed to differences in participant characteristics or intervention protocols. To clarify this issue, a future meta-analysis incorporating additional studies is warranted. Regarding the optimal duration of resistance training, Monsegue et al. [44] reported that high-intensity resistance training performed three times per week for at least eight weeks was most effective in improving muscle strength and function during recovery after TKA.

In terms of intervention type, a statistically significant subgroup difference was found for self-reported outcomes, with resistance training alone being more effective than when it was included as part of a multicomponent intervention. A similar tendency was observed for pain, strength, and performance-based function, although these differences did not reach statistical significance. A previous meta-analysis showed that, compared to resistance training alone, concurrent resistance and aerobic training was equally effective in increasing muscle mass in middle-aged and older adults [50]. Additionally, a meta-analysis targeting patients with knee osteoarthritis found that a single type of exercise was more effective than a combined exercise program that included resistance, aerobic, and functional training [51]. This may be due to training time and effort being divided among multiple components, potentially diluting the neuromuscular stimulus of resistance training. Another possible explanation is the interference effect. In this study, the multicomponent interventions included flexibility training and functional tasks such as toe raises and step exercises. Although these components do not constitute endurance training per se, their repetitive and low-intensity nature may resemble endurance-type activity. Prior research suggests that combining endurance and resistance training can attenuate muscular adaptations compared to resistance training alone, indicating the possibility that similar interference effects may have occurred in the multicomponent groups [52]. Wilson et al. [53] reported that performing endurance and strength training simultaneously may inhibit maximum power and rate of force development due to interference effects. These findings align with the trends observed in the present study and suggest that the proportion of resistance training within multicomponent protocols could potentially influence the outcomes. However, given the largely exploratory nature of these subgroup findings, cautious interpretation is warranted, and further studies are required to confirm these outcomes.

This meta-analysis has several limitations. First, the number of studies included was relatively small compared to previous meta-analyses. This was primarily due to the exclusion of studies that initiated interventions or measured baseline outcomes prior to surgery, as the present analysis focused exclusively on the effects of resistance training after TKA. These criteria allowed for the analysis of the isolated effects of resistance training following TKA surgery. However, preoperative age and WOMAC pain and function scores should be considered collectively due to their impact on postoperative prognosis [54]. Second, as reported in previous meta-analyses [11,12,13], overall heterogeneity was high. This likely stems from differences in subject characteristics, intervention methods, and training intensity. Future studies should be conducted to clearly evaluate the effectiveness of resistance training after TKA surgery. These studies should be high-quality RCTs that standardize the components of the interventions and minimize dropout rates and missing data. Third, this review did not include a search of grey literature, which may have resulted in the omission of unpublished studies. Furthermore, data collection may have been incomplete, as no attempts were made to contact study authors when full texts were unavailable.

## 5. Conclusions

This meta-analysis suggests that resistance training may contribute to improvements in pain, muscle strength, and function among patients with TKA. Furthermore, it was observed that the intervention tended to be more effective when the duration was less than 12 weeks and when resistance training was applied alone. These findings may help inform the design of resistance training protocols in clinical settings. Future studies should be high-quality RCTs with standardized intervention timing, intensity, and composition.

## Figures and Tables

**Table 1:** Subgroup analysis of the effects of intervention duration and type.

Outcome	Subgroup	Study		Effect Size (SMD [95% CI])	I^2^	Test for Subgroup Difference
Pain	Comparison group	[19,20,21,22,24]	Other intervention	1.08 [0.20; 1.97]	89.8%	*p* = 0.023
		[23,25]	No intervention	−0.01 [−0.33; 0.31]	0%	
	Intervention Duration	[19,21,24]	~11 weeks	1.38 [0.02; 2.74]	92.6%	*p* = 0.199
		[20,22,23,25]	12 weeks~	0.39 [−0.26; 1.04]	77.2%	
	Intervention type	[19,20,21,22,25]	Resistance only	1.05 [0.14; 1.96]	90.2%	*p* = 0.101
		[23,24]	Multicomponent	0.16 [−0.39; 0.71]	66.8%	
Muscle strength	Comparison group	[20,24]	Other intervention	1.52 [0.59; 2.46]	77.4%	*p* = 0.022
		[23,25]	No intervention	0.32 [−0.10; 0.75]	34.4%	
	Intervention Duration	[20,23,25]	~11 weeks	1.12 [0.14; 2.10]	87.1%	*p* = 0.580
		[24]	12 weeks~	0.80 [0.28; 1.33]	-	
	Intervention type	[20,25]	Resistance only	1.48 [0.43; 2.52]	80.4%	*p* = 0.101
		[23,24]	Multicomponent	0.46 [−0.17; 1.08]	73.5%	
Self-reported function	Comparison group	[19,20,21,22,24]	Other intervention	1.77 [0.15; 3.38]	93.9%	*p* = 0.102
		[25]	No intervention	0.33 [−0.25; 0.92]	-	
	Intervention Duration	[19,21,24]	~11 weeks	2.05 [−0.79; 4.90]	96.3%	*p* = 0.542
		[20,22,25]	12 weeks~	1.10 [−0.02; 2.22]	84.7%	
	Intervention type	[19,20,21,22,25]	Resistance only	1.80 [0.22; 3.39]	93.3%	*p* = 0.041
		[24]	Multicomponent	0.06 [−0.44; 0.57]	-	
Performance-based function	Comparison group	[19,20,21,22,24]	Other intervention	0.92 [0.53; 1.31]	59.2%	*p* = 0.011
		[23,25]	No intervention	0.27 [−0.05; 0.59]	0%	
	Intervention Duration	[19,21,24]	~11 weeks	1.14 [0.60; 1.68]	66.9%	*p* = 0.014
		[20,22,23,25]	12 weeks~	0.39 [0.14; 0.65]	0%	
	Intervention type	[19,20,21,22,25]	Resistance only	0.81 [0.34; 1.28]	72.6%	*p* = 0.345
		[23,24]	Multicomponent	0.52 [0.13; 0.91]	34.4%	

SMD: Standardized mean difference; CI: Confidence interval.

## Data Availability

Data can be requested from the corresponding author and will be released on reasonable request.

## Supplementary Materials

The following supporting information can be downloaded at: https://www.mdpi.com/article/10.3390/jcm14144979/s1, Table S1: Search strategy; Table S2: List of excluded studies; Table S3: Characteristics of studies included in the systematic review; Table S4: Certainty of evidence; Figure S1: Risk of bias in studies.

## Abbreviations

The following abbreviations are used in this manuscript:

OA	Osteoarthritis
TKA	Total Knee Arthroplasty
RCTs	Randomized Clinical Trials
BMI	Body Mass Index
WOMAC	Western Ontario and MacMaster Universities Osteoarthritis Index
KOOS	Knee Injury and Osteoarthritis Outcome Score
VAS	Visual Analogue Scale
ADL	Activities of Daily Living
HSS	Hospital for Special Surgery
ROB 2	Risk of Bias 2
SMD	Standardized Mean Difference
CIs	Confidence Intervals
1RM	One Repetition Maximum
