# Supplementary material for: Effects of Resistance Training on Pain, Muscle Strength, and Function in Patients Undergoing Total Knee Arthroplasty: A Systematic Review and Meta-Analysis
Source: J Clin Med. 2025 Jul 14;14(14):4979. doi: 10.3390/jcm14144979 (PMC12295842; doi:10.3390/jcm14144979)
Supplement: Supplementary file 1 [file jcm-14-04979-s001.zip › jcm-3724604-supplementary.pdf]

Table S1. Search strategy.

Database	Search strategy
Pubmed	#1 "Arthroplasty, Replacement, Knee"[Mesh]<br>#2 Arthroplasties, Replacement, Knee<br>#3 "Resistance Training"[Mesh]<br>#4 Resistance training<br>#5 Strength training<br>#6 #1 OR #2<br>#7 #3 OR #4 OR #5<br>#8 #6 AND #7
Web of science	(Total knee arthroplasty OR Total knee replacement) AND (Resistance Training OR Strength training)
Cochrane Library	#1 MeSH descriptor: [Arthroplasty, Replacement, Knee] explode all trees<br>#2 Arthroplasty, Replacement, Knee<br>#3 MeSH descriptor: [Resistance Training] explode all trees<br>#4 Resistance training<br>#5 Strength training<br>#6 #1 OR #2<br>#7 #3 OR #4 OR #5<br>#8 #6 AND #7
Embase	#1 ‘total knee arthroplasty’/exp<br>#2 ‘total knee arthroplasty’<br>#3 ‘resistance training’/exp<br>#4 ‘resistance training’<br>#5 ‘strength training’<br>#6 #1 OR #2<br>#7 #3 OR #4 OR #5<br>#8 #6 AND #7

Table S2. List of excluded studies

No	Author	Summary comment for exclusion
1.	Hold M [26]	Not English
2	Chen H [27]	Not English
3.	Vuorenmaa M [28]	No mean standard deviation
4.	Trudelle-Jackson E [29]	No mean standard deviation
5.	Husby VS [30]	No mean standard deviation
6.	Shabbir M [31]	The comparison group included resistance training.
7.	Fung V [32]	The comparison group included resistance training.
8.	Codine PH [33]	The comparison group included resistance training.
9.	Tanaka R [34]	The baseline measurement was taken before surgery
10.	Risso AM [35]	The baseline measurement was taken before surgery
11.	Mau-Moeller A [36]	The baseline measurement was taken before surgery
12.	Li Z [37]	The baseline measurement was taken before surgery
13.	Jakobsen TL [38]	The baseline measurement was taken before surgery
14.	Jacksteit R [39]	The baseline measurement was taken before surgery
15.	Jiao S [40]	Intervention began before surgery

Table S3. Characteristics of studies included in the systematic review.

Study	Patients			Intervention				Outcome
	Age: Mean (SD)	Sex(M/F)	BMI Mean (SD)	EG	CG	Session	Intensity	
Çetinkaya, 2022 [19]	(n= EG/CG)<br>60–65: 10/12<br>66-70: 15/12<br>71~: 5/6	Total: 60<br>EG: 3/27<br>CG: 4/26	-	Home exercise program + Elastic band: Knee flexion and extension movement and leg-lifting exercise.	Home exercise program incorporating knee flexion and extension exercises.	4 weeks, 4 times daily with at least 2 hours between applications.	Moderate intensity: excessive exertion avoided	Pain: VAS<br><br>Function: WOMAC total, 40-m brisk walking test
Hsu WH, 2019 [20]	EG: 72(1.8)<br>CG: 69.5(1.5)	Total: 29<br>EG: 0/14<br>CG: 0/15	EG: 29.4(1.5)<br>CG: 28.9(1)	Resistance training: Leg press machine, Leg extension machine, Seat leg curl machine, Hip adductor machine	Straight leg raises and range of motion exercises	24 weeks, 3 times per week	12 repetitions/set × 3 sets<br><br>1-4 week: 60% of 1RM<br><br>5-8: 70% of 1RM<br><br>9-24: 80% of 1RM	Pain: KOOS pain<br><br>Strength: Knee extension<br><br>Function: KOOS ADL, 8-ft Up-and go
Do K, 2020 [21]	EG1:72.84(7.03)<br>EG2: 72.5(4.73)<br>CG: 73.13(none)	Total: 55<br>EG1:3/16<br>EG2:3/17<br>CG:3/13	EG1: 37.19(2.59)<br><br>EG2: 37.6(3.03)<br><br>CG: 37.62(2.75)	EG1 - Hip exercise: Warm up (AROM), Supine extension bridge with thera-band, sideway walking with thera-band, standing hip adduction with thera-band, clamshell (Hip external	AROM	4 weeks, 3 times per week	EG1: 3 sets of 20 at a RPE 5~7<br><br>EG2: 4 sets of 20 at a RPE 5~7	Pain: WOMAC: pain<br><br>Function: WOMAC function, gait speed

				rotation) with thera-band<br><br>EG2 - Quadriceps exercise: warm up (AROM), seated knee extension with thera-band, supine straight leg raise with thera-band, quarter wall squat with thera-band				
Liao CD, 2020 [22]	EG: 72.22(7.75)<br>CG: 69.79(6.72)	Total: 40<br>EG: 0/20<br>CG: 0/20	EG: 28.27(3.25)<br>CG:27.6(3.64)	Elastic RET: knee extension, knee flexion, hip flexion, and hip extension	Standard care: Stretching exercise, ROM, cycling, treadmill	12 weeks, 2 times per week	Three sets of 10–20 repetitions	Pain: WOMAC pain<br><br>Function: WOMAC function, gait speed
Heikkilä A, 2017 [23]	EG: 69(8)<br>CG: 69(9)	Total: 135<br><br>EG: 23/57<br><br>CG: 19/36	EG:31(5)<br>CG: 31(6)	Progressive home exercise program: isometric strengthening exercises for the quadriceps and hamstring muscles, squats, flexibility exercises, step exercises; functional exercises: rising on the toes, first on both legs and on one leg, step exercises	No additional guidance was provided after discharge from the hospital.	1-2 times per day for 1 year	10-15 repetitions	Pain: Knee pain during loading<br><br>Strength: Knee extension<br><br>Function: velocity maximal
Unver B, 2016 [24]	EG: 69.53(9.2)<br>CG: 69.8(5.3)	Total: 60<br>EG: 6/24<br>CG: 4/26	EG:31.82(6.05)<br>CG: 32.17(6.2)	Weighted treatment: 12 basic isometric, active resisted range of motion, various resisted straight	Non-weighted treatment	8 weeks, 1 time per week	All exercises were progressed to 15 repetitions.	Pain: activity pain,<br><br>Strength:

				leg raising exercises, and static stretching exercises for the lower limbs.				quadriceps muscle<br><br>Function: HSS knee scores, 10m walk test
Valtonen A, 2010 [25]	EG: 66.2(6.3)<br>CG: 65.7(6)	Total: 60<br>EG: 10/26<br>CG: 14/10	Weight<br>EG: 83.2(15.2)<br>CG: 83.9(15)<br><br>Height<br>EG: 167.3(9.3)<br>CG: 169.7(8.2)	Aquatic resistance: knee extension-flexion movement in a sitting position, hip abduction-adduction with extended knee in a standing position, hip extension-flexion with extended knee in a standing position, knee extension-flexion in a standing position, and step-squat backward from the aqua aerobic step board.	No interventi on	12 weeks ,2 times per week	Perceived Exertion scale	Pain: WOMAC pain<br><br>Strength: Knee extension power<br><br>Function: WOMAC physical function, maximal walking speed

EG: experimental group; CG: Control group; VAS: Visual analogue scale; WOMAC: The Western Ontario and McMaster Universities Arthritis Index; RM: repetition maximum; KOOS: Knee Injury and Osteoarthritis Outcome Score; ADL: Activities of daily living; AROM: Active range of motion; RPE: Rate of perceived exertion' RET: Resistance exercise training; ROM: Range of motion; HSS: Hospital for special surgery.

Table S4. Certainty of evidence

Outcomes	Studies and participants	Risk of bias	Inconsistency	Indirectness	Imprecision	Publication bias	Effect size (95% CI)	I^2	Evidence level
Pain	7 studies, 443	Serious	Serious	Not Serious	Not Serious	Cannot determine	0.84 [0.11; 1.57]	89.6%	Moderate
Strength	4 studies, 269	Serious	Serious	Not Serious	Not Serious	Cannot determine	1.03 [0.29; 1.77]	83.1%	Moderate
Self-reported function	6 studies, 335	Serious	Very Serious	Not Serious	Serious	Cannot determine	1.58 [0.15; 3.01]	93.1%	Low
Performance-based function	7 studies, 443	Serious	Not Serious	Not Serious	Not Serious	Cannot determine	0.74 [0.38, 1.11]	68.9%	Moderate

CI: Confidence Intervals

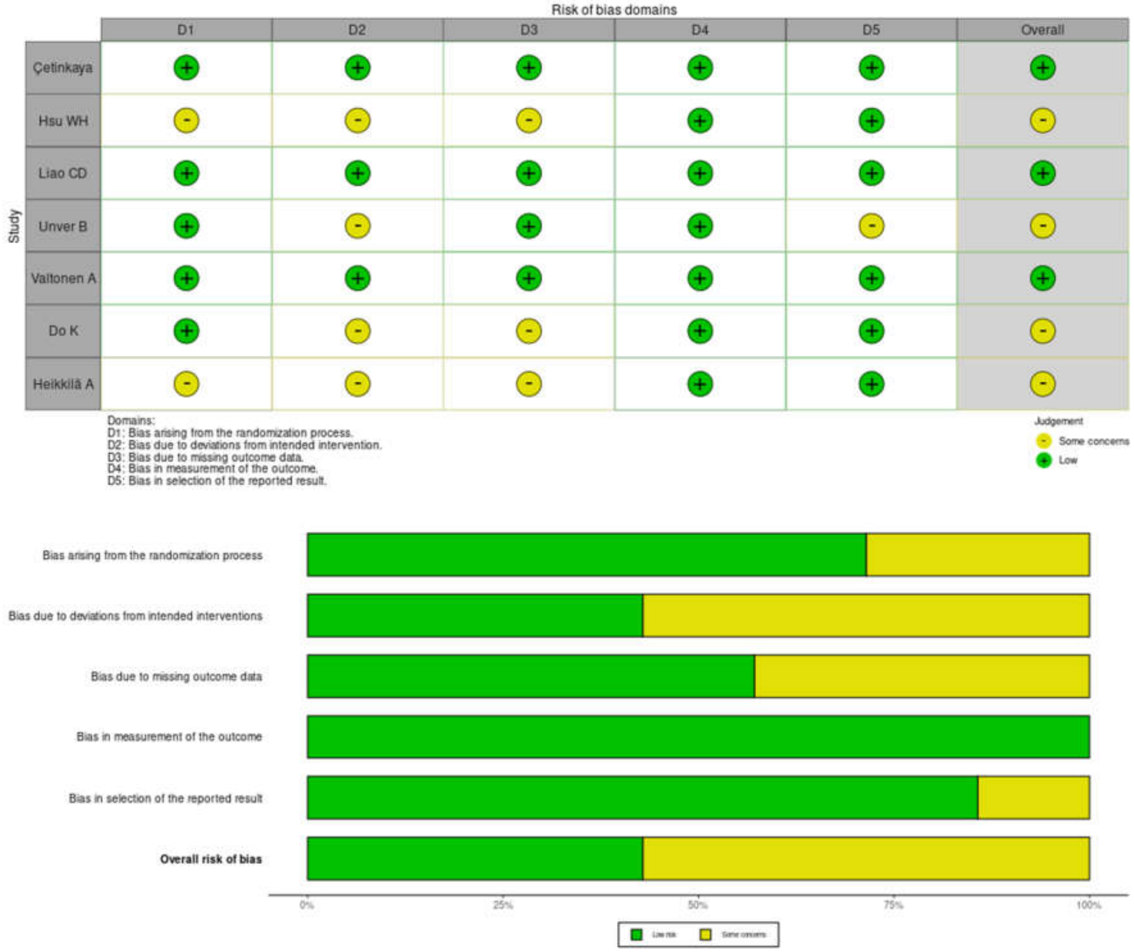

Figure S1. Risk of bias in studies.
